# Supplementary material for: Inflammatory Bowel Disease Guidelines for Corneal Refractive Surgery Evaluation
Source: J Clin Med. 2022 Aug 19;11(16):4861. doi: 10.3390/jcm11164861 (PMC9409909; doi:10.3390/jcm11164861)
Supplement: Supplementary file 1 [file jcm-11-04861-s001.zip › jcm-1851080-supplementary.pdf]

Table S1: Preoperative and Postoperative Measurements

Patient	Eye	Procedure	Preop K1	Preop K2	Preop Pachymetry	Ablation Depth	Residual Stromal Thickness	Preop UDVA	Preop MRx	Preop BDVA	1 year UDVA	1 year MRx	1 year BDVA
1	OD	SMILE w Zeiss VisuMax	44.6 D @ 177	45.7 D @ 87	559 µm	[a]107/15 µm	335 µm	CF	-5.50	20/20	[b]20/20-1	[b]+0.50 Sphere	[b]20/20
1	OS	SMILE w Zeiss VisuMax	44.8 D @ 164	45.6 D @ 74	557 µm	[a]96/15 µm	343 µm	20/600	-4.50 -0.25 @ 176	20/20	[b]20/20-2	[b]+0.50 -0.50 @005	[b]20/20
2	OD	PRK w WaveLight EX500	42.3 D @ 70	43.6 D @ 160	547 µm	52 µm	440 µm	20/250	-2.00 -1.50 @ 081	20/20	20/20	.0.25 Sphere	20/20
2	OS	PRK w WaveLight EX500	42.7 D @ 105	43.5 D @ 15	539 µm	72 µm	415 µm	20/500	-4.00 -1.25 @ 090	20/20	20/20	Plano -0.50 @180	20/20
3	OD	LASIK w WaveLight EX500 and Zeiss VisuMax	42.9 D @ 2	44.3 D @ 92	538 µm	33 µm	399 µm	20/150	-1.75 -0.75 @ 175	20/20	[c]20/15	[c]+0.25 Sphere	[c]20/20
4	OD	LASIK w WaveLight EX500 and Zeiss VisuMax	44.9 D @ 85	45.5 D @ 175	532 µm	47 µm	382 µm	20/500	-2.50 -0.75 @ 095	20/20	20/20-1	Plano -0.50 @176	20/20
4	OS	LASIK w WaveLight EX500 and Zeiss VisuMax	44.6 D @ 75	45.6 D @ 165	544 µm	37 µm	402 µm	20/200	-1.50 -1.25 @ 074	20/20-1	20/25-1	-0.25 -0.25 @013	20/20
5	OD	LASIK w WaveLight EX500 and Zeiss VisuMax	43.5 D @ 10	44.6 D @ 100	570 µm	49 µm	420 µm	20/250	-2.50 -0.25 @ 005	20/20	20/20	Plano Sphere	20/20
5	OS	LASIK w WaveLight EX500 and Zeiss VisuMax	43.6 D @ 168	44.5 D @ 78	569 µm	42 µm	416 µm	20/400	-2.50 -0.50 @ 012	20/20	20/20	+0.25 Sphere	20/20
6	OD	LASIK w WaveLight EX500 and Zeiss VisuMax	42.7 D @ 24	43.3 D @ 114	535 µm	56 µm	375 µm	20/300	-3.25 -0.50 @ 069	20/20	20/15	+0.25 Sphere	20/15
6	OS	LASIK w WaveLight EX500 and Zeiss VisuMax	42.6 D @ 149	44.4 D @ 59	539 µm	68 µm	368 µm	20/300	-3.25 -1.25 @139	20/20	20/20	Plano -0.75 @160	20/20 + 3
7	OD	LASIK w WaveLight EX500 and WaveLight FS200	44.8 D @ 175	45.7 D @ 85	551 µm	78 µm	366 µm	20/250	-5.00 -0.25 @135	20/20	20/20	Plano Sphere	20/20
7	OS	LASIK w WaveLight EX500 and WaveLight FS200	45.1 D @ 5	46.0 D @ 95	552 µm	88 µm	364 µm	20/500	-5.50 sph	20/20	20/20-1	PL -0.25 @ 165	20/20-1
8	OD	LASIK w WaveLight EX500 and WaveLight FS200	45.4 D @ 134	45.6 D @ 44	612 µm	50 µm	455 µm	20/400	-3.00 -0.75 @ 106	20/20	[b]20/20	[b]+0.25 Sphere	[b]20/20
8	OS	LASIK w WaveLight EX500 and WaveLight FS200	45.3 D @ 148	45.4 D @ 58	523 µm	28 µm	392 µm	20/250	-2.75 -0.50 @ 084	20/20	[b, d]20/70	[b, d]-1.25 -0.50 @ 002	[b]20/20
9	OD	LASIK w WaveLight EX500 and Zeiss VisuMax	44.7 D @ 170	46.0 D @ 80	561 µm	44 µm	403 µm	20/400	-2.75 Sphere	20/20	20/25-1	+0.75 -0.25@118	20/20
9	OS	LASIK w WaveLight EX500 and Zeiss VisuMax	44.5 D @ 9	45.3 D @ 99	555 µm	44 µm	397 µm	20/500	-2.75 Sphere	20/20	20/20-1	Plano -0.50@66	20/20
10	OD	LASIK w WaveLight EX500 and Zeiss VisuMax	45.2 D @169	46.0 D @ 79	534 µm	45 µm	388 µm	20/125	-3.00 Sphere	20/20	20/15-1	Plano Sphere	20/15
10	OS	LASIK w WaveLight EX500 and Zeiss VisuMax	45.6 D @ 6	46.6 D @ 96	540 µm	30 µm	405 µm	20/200	-2.00 Sphere	20/20	20/15	Plano Sphere	20/15
11	OD	LASIK w WaveLight EX500 and Zeiss VisuMax	44.7 @ 174	45.6 @ 84	534 µm	69 µm	359 µm	20/500	-4.75 -0.25 @ 090	20/20	[e]20/25+	[f]N/A	[f]N/A
11	OS	LASIK w WaveLight EX500 and Zeiss VisuMax	44.6 D @ 18	45.6 D @ 108	530 µm	66 µm	359 µm	20/500	-4.50 -0.25 @ 045	20/20	[e]20/20+	[f]N/A	[f]N/A

UDVA: Uncorrected Distance Visual Acuity; MRx: Manifest Refraction; BDVA: Best Corrected Distance Visual Acuity; OD: Right Eye; OS: Left Eye

[a]Removed lenticule thickness at center/periphery; [b]3.5 months follow-up value; [c]4 months follow-up value; [d]Eye was corrected for monovision: target -1.25 Sphere; [e]1 week follow-up value; [f]Not available due to no data secondary to short follow-up
